# Myeloid *Vamp3* deletion attenuates CFA-induced inflammation and pain in mice via ameliorating macrophage infiltration and inflammatory cytokine production

**DOI:** 10.3389/fimmu.2023.1239592

**Published:** 2023-10-27

**Authors:** Xiaolong Dai, Lianlian Li, Xinrong Yan, Qianqian Fan, Ruizhen Wang, Wenhao Zhang, Weiwei Chen, Yang Liu, Jianghui Meng, Jiafu Wang

**Affiliations:** ^1^ School of Life Sciences, Henan University, Kaifeng, China; ^2^ School of Biotechnology, Faculty of Science and Health, Dublin City University, Dublin, Ireland

**Keywords:** vesicle associated membrane protein, inflammation, cytokine, rheumatoid arthritis, pain, SNARE, therapeutics, neuropeptides

## Abstract

Persistent inflammation and associated pain significantly impact individuals’ quality of life, posing substantial healthcare challenges. Proinflammatory cytokines, released by activated macrophages, play crucial roles in the development of chronic inflammatory conditions such as rheumatoid arthritis. To identify and evaluate potential therapeutic interventions targeting this process for mitigating inflammation and pain, we created myeloid cell-specific knockout of *Vamp3* (vesicle-associated membrane protein 3) mice (*Vamp3* ^Δmyel^) by crossing *LysM-Cre* mice with newly engineered *Vamp3^flox/flox^* mice. Bone marrow-derived macrophages and peritoneal resident macrophages from *Vamp3* ^Δmyel^ mice exhibited a significant reduction in TNF-α and IL-6 release compared to control mice. Moreover, Vamp3 deficiency led to decreased paw edema and ankle joint swelling induced by intraplantar injection of complete Freund’s adjuvant (CFA). Furthermore, Vamp3 depletion also mitigated CFA-induced mechanical allodynia and thermal hyperalgesia. Mechanistically, Vamp3 loss ameliorated the infiltration of macrophages in peripheral sites of the hind paw and resulted in reduced levels of TNF-α and IL-6 in the CFA-injected paw and serum. RT-qPCR analysis demonstrated downregulation of various inflammation-associated genes, including *TNF-α*, *IL-6*, *IL-1β*, *CXCL11*, *TIMP-1*, *COX-2*, *CD68*, and *CD54* in the injected paw at the test day 14 following CFA administration. These findings highlight the novel role of Vamp3 in regulating inflammatory responses and suggest it as a potential therapeutic target for the development of novel Vamp-inactivating therapeutics, with potential applications in the management of inflammatory diseases.

## Introduction

Chronic inflammation, lasting from several months to years, significantly impacts individuals’ well-being, resulting in enduring pain and reduced mobility. Cytokines play a pivotal role in the intricate web of signaling molecules that regulate this condition. Proinflammatory cytokines are often overproduced or dysregulated in chronic inflammation, leading to a sustained immune response. They serve as conductors for immune cell activation, amplification of inflammatory processes, and modulation of neighboring cell behavior (1). Noteworthy examples include tumor necrosis factor-alpha (TNF-α), interleukin-1 beta (IL-1β), and IL-6, which are often elevated in disorders like rheumatoid arthritis (RA) (2–4), intensifying inflammation and contributing to tissue damage. While conventional anti-inflammatory drugs provide temporary relief, they are primarily palliative. Although biological drugs targeting specific cytokines or receptors have shown promise (5, 6), their high cost, side effects, and limited efficacy emphasize the urgent need for improved versions targeting new mechanisms.

Monocyte/macrophage lineage cells are major sources of cytokines and enzymes. In inflamed synovium, activated macrophages release inflammatory mediators and MMPs that contribute to joint destruction (7). Chemicals produced due to tissue injury, such as TNF-α, IL-1, prostaglandins, bradykinin, H^+^, histamine, and ATP, bind to their respective receptors, generating action potentials in the peripheral terminals of C- and A-δ sensory fibers in the injured joint (8–10), leading to central pain sensation. This, in turn, elicits reflex antidromic efferent signals that travel back from the central to the articular tissues. This process triggers the release of local pain mediators such as glutamate, substance P (SP), and calcitonin gene-related peptide (CGRP) (10, 11). These molecules can modulate macrophages, synoviocytes, and T cells, and augment the production of pro-inflammatory cytokines, prostaglandin E2, and collagenase (10, 12–14). This feed-forward loop results in persistent and pathological inflammation. TNF-α and IL-6 directly contribute to mechanical hyperalgesia by sensitizing joint nociceptors to mechanical stimulation (15, 16), while IL-1β and its receptor contribute to thermal hyperalgesia in arthritic knee pain (17). Understanding and targeting these cytokine pathways hold great promise for the development of more effective therapies aimed at alleviating pain and modulating chronic inflammation.

Earlier studies have shown that TNF-α and IL-6 intracellular trafficking to recycling endosomes and secretion from cultured macrophages require vesicle-associated membrane protein 3 (Vamp3), a vesicular-SNARE (soluble N-ethylmaleimide-sensitive factor attachment protein receptors) (18–20). SNAREs are involved in vesicular trafficking, membrane fusion, vesicle release, and replenishment of membrane receptors/channels (21). Vamp3 interacts with targeted SNARE partners to form SNARE complexes, driving the fusion of cytokine-containing vesicles with the plasma membrane and the subsequent release of cytokines from human synovial sarcoma cells (22). However, the impact of Vamp3 deficiency on inflammation and pain remains elusive. Complete Freund’s adjuvant (CFA) has been employed in various animal models to elicit inflammation and investigate inflammatory pain (23, 24). Given the prominent role of monocyte/macrophage lineage cells, we hypothesized that Vamp3 deficiency in myeloid cells would attenuate the severity of CFA-induced inflammation. To address this question, we created a myeloid *Vamp3* deletion mouse model (*Vamp3^Δmyel^)*, which reduced the release of TNF-α and IL-6 from macrophages. Notably, myeloid *Vamp3* deletion reduced CFA-induced inflammation and pain, highlighting that Vamp3 might be an attractive target for the treatment of certain chronic inflammatory conditions.

## Materials and methods

### Production of *Vamp3^Δmyel^* mice

Conditional knockout *Vamp3^flox/flox^* mice on a C57BL/6N background were custom-designed and generated by Cyagen Biosciences Inc. (Taicang, China) based on the CRISPR/Cas-mediated genome engineering. To engineer the targeting vector, mouse genomic fragments containing homology 5’ arm (1595 bp) and 3’ arm (1589 bp) and conditional knockout region (1881 bp) were generated by PCR using Bacterial Artificial Chromosome clone RP23-35E21 and subsequentially assembled into the SCK plasmid. Two loxP recombination sites were inserted into the introns flanking the essential coding exons 3 and 4 to yield a targeting vector SCK004-VT. The knockout of exons 3 and 4 resulted in a frameshift of the gene. Cas9, gRNA, and targeting vector were co-injected into fertilized eggs for *Vamp3^flox/flox^* mouse production. The pups were genotyped by PCR followed by sequencing analysis. The resultant *Vamp3^flox/flox^* mice were bred with the *LyzM-Cre* mice (sourced from Cyagen Biosciences Inc.) bearing the Cre recombinase inserted in the *lysozyme 2* gene locus (25) to yield the mice with myeloid cell-specific deletion of *Vamp3* (*Vamp3* ^Δmyel^). The genotypes of all the mice were confirmed using PCR with the primers listed in **Supplementary Figure 1**. Additionally, Western blotting was performed to confirm the absence of Vamp3 expression in the isolated macrophages. Female *Vamp3* ^Δmyel^ mice aged ~8 weeks were used for all behavioral experiments. Age-matched female offspring from *LysM-Cre* mice breeding were used as wild-type (WT) controls. All mice were bred and housed in an approved Bioresource Unit at Henan University, with 4 or 5 animals per cage. They had free access to food and water and were kept under controlled conditions of humidity (~50%), temperature (22°C), and a 12-hour light/dark cycle.

### CFA-induced mouse inflammation model and histological assessment

A mouse inflammation model was established by intraplantar injection of 15 μl of 1 mg/mL CFA (Merck, Shanghai, China, catalog number F5881) into the right hind paw of *Vamp3* ^Δmyel^ and WT mice. The injected mice were then randomized into different groups. The behavioral studies were conducted with the tester remaining unaware of the identity of the mice until the completion of the experiment. Paw edema and ankle diameter at various times were measured using digital calipers. For isolation of tissues for histopathological examination, some mice were deeply anesthetized with an intraperitoneal injection of sodium pentobarbitone (60 mg/kg body weight) (Sinopharm Chemical Reagent Co., Ltd., Shanghai, China) at day 1 and day 14 post CFA injection. The isolated plantar skin was fixed with 4% paraformaldehyde and processed through dehydration, wax leaching, embedding, and cutting into 4-μm thickness slices by Servicebio Technology Co., Ltd. (Wuhan, China). Following these steps, the slices were subjected to hematoxylin and eosin (H&E) staining. Epidermal thickness was measured in 5 randomly selected fields of view per mouse using CellSens Dimension Imaging software. The extent of dermal inflammation was semiquantified using a 0 to 4 scoring system (0, no inflammation; 1, little; 2, mild; 3, moderate; 4, severe) by Servicebio Technology.

Immunohistochemical analysis of CD68 expression was conducted by Servicebio Technology. Briefly, skin sections from CFA-injected paws were deparaffinized using xylene and then rehydrated through a series of graded ethyl alcohols and distilled water. Heat-mediated antigen retrieval was performed, followed by blocking endogenous peroxidase activity with a peroxidase-blocking reagent (3% H_2_O_2_). The samples were permeabilized in PBS with 0.3% Triton X-100 and then incubated in a blocking solution (PBS with 3% bovine serum albumin) for 1 hour. Subsequently, the specimens were incubated overnight at 4°C with a rabbit antibody against mouse CD68 (Servicebio Technology, catalog number GB113109, 1:200) in the blocking solution. After three washes with PBS, the samples were further incubated with horseradish peroxidase-conjugated goat anti-rabbit secondary antibody (Servicebio Technology, catalog number GB23303, 1:200) at room temperature for 1 hour. Excess unbound antibodies were washed away, and the specimens were developed using a DAB chromogenic substrate kit (Servicebio Technology, catalog number G1212) and counterstained with hematoxylin before dehydration and mounting a coverslip onto the slide. Images were taken using an IX73 Olympus inverted microscope and analyzed with CellSens Dimension Imaging software.

### Measurement of mechanical and heat hyperalgesia

Before measuring the mechanical withdrawal threshold, mice were placed in individual compartments with mesh floors and allowed to habituate for at least 20 min. Mechanical sensitivity was evaluated using standard von Frey filaments (Ugo Basile, Italy, catalog number NC12775) and 50% thresholds were calculated using the up-down technique (26). The von Frey hairs used covered a range of bending forces (0.04-8 g).

Thermal hyperalgesia was assessed using the Hargreaves method (27). Briefly, mice were habituated in enclosures for 20 min. An infrared beam was placed under the plantar surface of each hind paw and the latency to withdraw was recorded as the time for paw withdrawal. A 20 s cutoff was set to avoid damage to the paw.

### Animal mobility

The spontaneous exploratory behavior of WT and *Vamp3* ^Δmyel^ mice was monitored using the Noldus EthoVision XT video tracking system (Noldus Information Technology, Wageningen, The Netherlands) (28). Briefly, an individual mouse was placed in a chamber of dimensions 20x20 cm and acclimated in the enclosures for 30 min for three consecutive days. On the experimental day, mice were placed in the chambers and locomotor activity was video-recorded for 20 min. The traveled distance during each test day was determined using the EthoVision XT system.

### Measurement of cytokine protein expression in the CFA-injected paws

The levels of proinflammatory cytokines TNF-α and IL-6 in the CFA-injected paws were measured following previously described protocols (23), with minor modifications. Briefly, the injected paws (including skin and underlying muscle) at day 1 and day 14 post CFA administration were dissected and homogenized on ice in radioimmunoprecipitation assay (RIPA) lysis and extraction buffer (Thermo Fisher Scientific, Shanghai, China, catalog number 89900) containing a protease inhibitor cocktail set III (1:100 v/v; Merck, catalog number 539134) and 1 mM phenylmethylsulfonyl fluoride (Merck, catalog number P7626) before centrifugation at 13,000 rpm for 10 min at 4°C. The supernatant was collected and stored at -80°C. The protein concentration in the supernatant was quantified using the Bradford reagent (Merck, catalog number B6916). The concentration of each sample was adjusted to 15 mg/ml. Aliquots of 100 μl were utilized to assess the cytokine concentrations using ELISA kits (R&D Systems, Bio-techno, Shanghai, China) for TNF-α (catalog number MTA00B) and IL-6 (catalog number M6000B), following the manufacturer’s instructions.

### Reverse transcription-quantitative real-time PCR

Murine plantar skins were harvested at day 14 post CFA injection and total RNA was extracted using an RNeasy Mini Kit (Qiagen, Hilden, Germany, catalog number 74134) following the manufacturer’s instructions. Total RNA (1 µg) was reverse transcribed into cDNA using a high-capacity cDNA reverse transcription kit (Thermo Fisher Scientific, catalog number 4368813). qPCR was performed by incubating cDNAs with the PowerUp™ SYBR™ Green Master Mix (Thermo Fisher Scientific, catalog number A25742) and primers (**Supplementary Table 1**) with normalization to GAPDH.

### Culturing macrophages and cytokine release assay

Peritoneal resident macrophages from approximately 7-week-old *Vamp3* ^Δmyel^ mice and WT mice were isolated following the protocol described by Zhang et al. (29). The peritoneal fluid was collected and centrifuged at 400 g for 10 minutes. The resulting cell pellet was then suspended in a culture medium consisting of DMEM/Nutrient F12 ham (Merck, catalog number D6429), 10% (v/v) FBS (Merck, catalog number 341506), and 1% (v/v) penicillin-streptomycin (Merck, catalog number V900929). Subsequently, 4x10^5^ cells were seeded per well in a 24-well plate. After a 2-hour incubation at 37°C with 5% CO_2_, the cells were washed twice with warmed PBS to eliminate non-adherent cells. Fresh culture medium was added, and the cells were further incubated for 24 hours. To stimulate the macrophages, LPS (100 ng/ml) (Merck, catalog number L4391) and INF-γ (500 pg/ml) (Merck, catalog number IF005) were added and incubated for 6 hours. The supernatant was then collected for TNF-α (catalog number MTA00B, R&D Systems, Bio-techno) and IL-6 (catalog number M6000B) concentration measurement using ELISA.

Mouse pelvic and femoral bones were used to isolate bone marrow according to a published protocol (30). After eliminating erythrocytes using red blood cell lysing buffer, the bone marrow cells were cultured for 7 days in the presence of 10 ng/ml macrophage colony-stimulating factor (Abcam, Shanghai, China, catalog number ab256080). The culture medium was refreshed every 2 days. Subsequently, the adherent cells were detached from the culture surface using a cell scraper, then resuspended in culture medium and seeded at a density of 1x10^5^ cells per well in a 24-well plate. After 24 hours, the macrophage cells were stimulated with 100 ng/ml LPS and 500 pg/ml INF-γ at 37°C for 6 hours to induce cytokine release.

### SDS-PAGE and Western blotting

The Western blotting assay was employed to confirm the Vamp3 protein knockout in macrophages derived from *Vamp3* ^Δmyel^ mice. After stimulation with LPS and INF-γ, the macrophages were solubilized in 60 µl of 2x LDS sample buffer (Thermo Fisher, catalog number B0007) and heated to 100°C for 10 minutes before undergoing SDS-PAGE using pre-cast NuPAGE 12% Bis-Tris gels (Thermo Fisher, catalog number NP034B). The separated proteins were transferred onto polyvinylidene difluoride membranes using electrotransfer. Following blocking with 5% BSA in TBST buffer, the membrane was bisected between the pre-stained markers at 33kDa and 25kDa. The lower portion was then incubated overnight at 4°C with a rabbit anti-Vamp1-3 pan antibody (Synaptic Systems, catalog number 104104, 1:1000). Note that Vamp1 and Vamp2 were barely detected in macrophages. To monitor protein loading, the upper portion of the membrane was subjected to overnight incubation at 4°C with a mouse monoclonal antibody against β-actin (Merck, catalog number A5316, 1:2000). Following extensive washing, the membranes were incubated with horseradish peroxidase-conjugated secondary antibodies specific to the respective species for 1 hour. After further washing, the membranes were developed using a chemiluminescence reagent (Thermo Fisher, catalog number 32106), and the protein bands were analyzed using a gel documentation system (Syngene G: BOX Chemi XX9, Shanghai, China).

For measurements of IL-1β, COX-2, and CD54 protein levels in paw tissues injected with CFA in both WT and *Vamp3* ^Δmyel^ mice, the homologized tissue extracts (see above) underwent SDS-PAGE followed by Western blotting. The process was carried out in a similar manner, except for the use of specific antibodies: anti-IL-1β mouse monoclonal antibody (Servicebio, catalogue number GB122059-100, 1:500), anti-COX-2 rabbit polyclonal antibody (Servicebio, catalogue number GB11077-2-100, 1:1000), and anti-CD54 rabbit polyclonal antibody (Servicebio, catalogue number GB11106-100, 1:2000), respectively. The intensities of protein bands were quantified using Image J software.

### Statistical analysis

Statistical analysis and graphing were conducted using Prism 9 software (GraphPad Software, San Diego, California, USA). All data are expressed as means ± SEMs, with sample sizes (n) specified in the figure legends. For comparisons between two groups, statistical significance was determined using unpaired two-tailed Student’s t-test followed by Welch’s correction. A probability value of less than 0.05 was considered statistically significant. NS denotes non-significant results with p > 0.05, while * indicates p < 0.05, ** indicates p < 0.01, and *** indicates p < 0.001.

## Results

### Knockout of *Vamp3* greatly reduced cytokine secretion from macrophages

**Figure 1:**
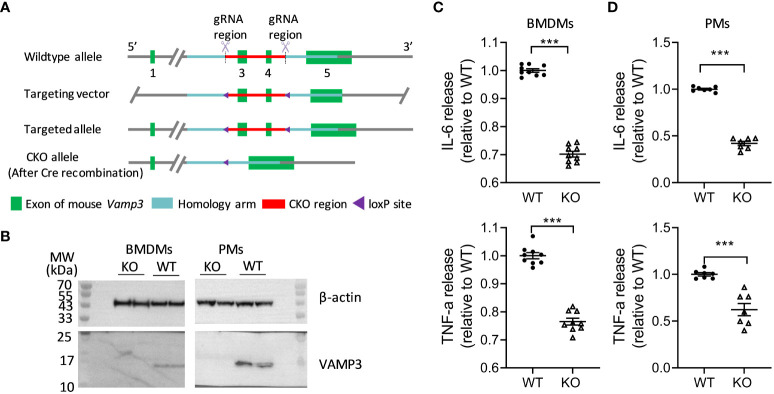
Effect of KO *Vamp3* on cytokine release from cultured macrophages. **(A)** Schematic representation of gene targeting method used in the generation of *Vamp3^flox/flox^* mice (details follow the Methods). **(B)** Western blots confirmed that cultured BMDMs and PMs prepared from *Vamp3* ^Δmyel^ mice did not express Vamp3. β-actin was probed as an internal loading control. Cultured BMDMs **(C)** and PMs **(D)** prepared from WT and *Vamp3* ^Δmyel^ mice were incubated with or without LPS and IFN-γ for 6 h before collection of the supernatant for quantification of the released TNF-α and IL-6 using their respective ELISA kits. Data are presented are mean ± SEM, n=3 in duplicates or triplicates. ***p<0.001, unpaired two-tailed Student’s t-test. BMDM, bone-marrow-derived macrophages; PM, peritoneal resident macrophages; WT, wild-type; KO, *Vamp3* ^Δmyel^.

To investigate the roles of Vamp3 in the inflammation, we generated a *Vamp3* conditional knockout mouse model (*Vamp3^flox/flox^*) in which the exons 3 and 4 of the *Vamp3* gene were flanked by loxP sites (**Figure 1A**). To achieve the specific deletion of *Vamp3* in myeloid cells, such as monocytes, macrophages, and granulocytes, *Vamp3^flox/flox^* mice were crossed with *LyzM-Cre* mice carrying the Cre recombinase inserted in the *Lysozyme 2* gene locus to generate myeloid-specific *Vamp3* knockout (*Vamp3* ^Δmyel^) mice. Mice deficient for the *Vamp3* allele in the myeloid cells did not display any gross phenotypic abnormalities in terms of development, growth, or survival. Efficient Cre-mediated recombination was detectable as revealed by the genotyping data (**Supplementary Figure 1**). To probe the role of Vamp3 in myeloid cells in mediating cytokine release, adherent bone-marrow-derived macrophages (BMDMs) and peritoneal resident macrophages (PMs) prepared from *Vamp3* ^Δmyel^ mice and WT controls were stimulated with LPS and INF-γ for 6 h before collecting the cells to probe the Vamp3 expression. Western blotting confirmed that Cre-mediated knockout resulted in a complete loss of the Vamp3 protein in cultured BMDMs and PMs from *Vamp3* ^Δmyel^ mice (**Figure 1B**). In comparison to the controls, loss of Vamp3 in BMDMs and PMs substantially reduced the release of TNF-α and IL-6 (**Figures 1C, D**). Our results demonstrated the importance of Vamp3 in mediating these pro-inflammatory cytokine releases from macrophages.

### CFA-induced mechanical and thermal hyperalgesia was attenuated in myeloid cell Vamp3 deficient mice

**Figure 2:**
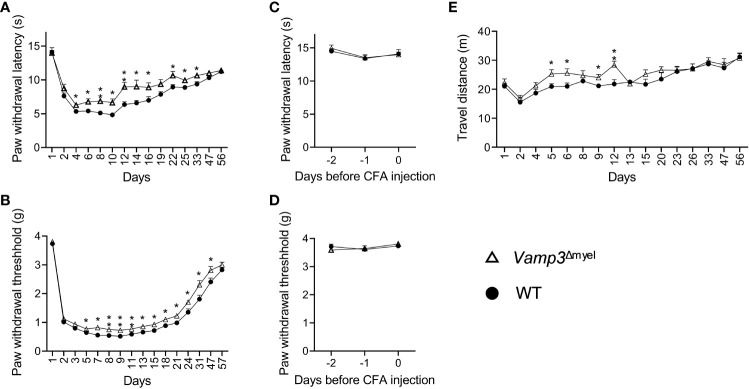
Effect of Vamp3 deficiency in myeloid cells on CFA-induced inflammatory pain. **(A, B)** Graphs show the time course of paw withdrawal latencies to a heat stimulus **(A)** and mechanical force required to elicit a withdraw response **(B)** in the CFA-injected right paw. CFA was injected on day 1 after measuring the baseline thresholds. **(C, D)** Plots indicate thermal latency **(C)** and mechanical withdrawal threshold **(D)** of the right paw prior to CFA injection. **(E)** Graph shows the traveled distance of WT and *Vamp3* ^Δmyel^ mice within 20 min after CFA injection. In some cases, the error bars are encompassed by the symbols. Data are mean ± SEM, n=8. *p < 0.05; **p < 0.01; unpaired two-tailed Student’s t-test.

There is abundant evidence showing that proinflammatory cytokines, such as TNF-α, IL-1β, and IL-6 are involved in the process of nociceptor sensitization and therefore contribute to persistent hyperalgesia [reviewed in (10)]. Thus, it is our particular interest to investigate whether myeloid cell Vamp3 depletion has any effect on attenuating inflammatory mediator-modulated inflammatory hyperalgesia. Towards this, a widely used mouse model of chronic inflammation was established in both *Vamp3* ^Δmyel^ mice and WT control mice following the intraplantar injection of CFA into the right hind paw. Inflammation induced by CFA was accompanied by significant mechanical allodynia and thermal hyperalgesia after measuring the mechanical withdrawal threshold and the thermal latency. CFA-injected control mice displayed a decreased thermal nociceptive threshold and a long-term reduction in mechanical thresholds (**Figures 2A, B**). Head-to-head comparisons of the nocifensive behaviors of *Vamp3* ^Δmyel^ mice were carried out with controls. No significant difference in basal mechanical withdrawal threshold and thermal latency was observed between *Vamp3* ^Δmyel^ mice and controls (**Figures 2C, D**), suggesting loss of myeloid Vamp3 does not affect the normal somatosensation. In contrast, *Vamp3* ^Δmyel^ mice showed a significant increment of the paw withdrawal latency to thermal stimulation than controls (**Figure 2A**). Similarly, the mechanical paw withdrawal threshold was significantly increased in *Vamp3* ^Δmyel^ mice vs controls (**Figure 2B**). Mouse spontaneous exploratory behavior was also monitored using the Noldus EthoVision XT video tracking system. Interestingly, there was a noticeable trend of increased spontaneous activity in *Vamp3* ^Δmyel^ mice compared to the control group during the first 2 weeks following CFA injection (**Figure 2E**).

### Myeloid *Vamp3* deletion attenuated CFA-induced inflammation

**Figure 3:**
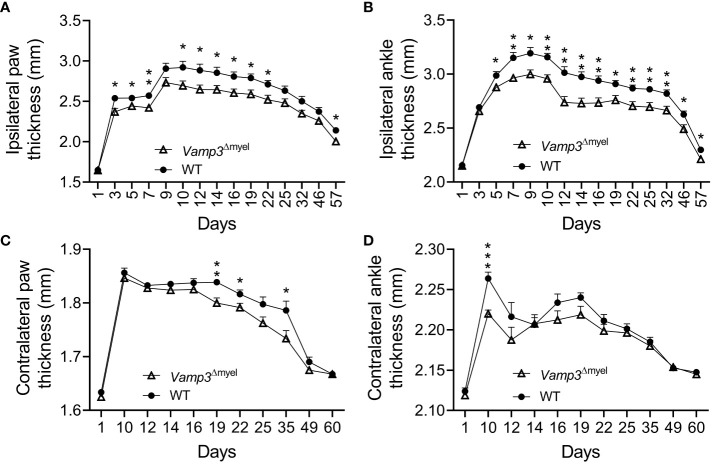
Effect of myeloid Vamp3 loss on CFA-induced inflammation. **(A-D)** Graphs show the thickness of the CFA-injected (ipsilateral) paw **(A)** and ankle **(B)** and the uninjected contralateral paw **(C)** and ankle **(D)** of WT and *Vamp3* ^Δmyel^ mice over time. CFA was injected on day 1 after measuring the thickness and basal thresholds. Data are mean ± SEM, n=8. *p < 0.05; **p < 0.01; ***p<0.001; unpaired two-tailed Student’s t-test.

Next, we examined the effect of Vamp3 depletion in myeloid cells on CFA-induced inflammation. After administering CFA, we observed significant swelling and noticeable redness in the injected paws of control mice during the initial week (see **Supplementary Figure 2**). By day 9, the average thickness of the hind paw had increased from 1.7 to 2.9 mm. CFA injection induced persistent paw swelling (**Figure 3A**). Similarly, CFA injection resulted in a significant increase in ipsilateral ankle joint diameter (**Figure 3B**). CFA injection indeed increased foot thickness and joint diameter on the contralateral side, but much less severe than the ipsilateral side (**Figure 3C** vs 3A and **Figure 3D** vs 3B). To determine whether loss of Vamp3 reduces CFA-induced inflammation, we compared the *Vamp3* ^Δmyel^ mice with control mice. Notably, KO *Vamp3* in myeloid cells significantly reduced the foot thickness at day 3 after CFA injection (**Figure 3A**). This effect lasted 8 weeks (**Figure 3A**). Consistently, ankle joint swelling was also attenuated by depletion of Vamp3 over 8 weeks (**Figure 3B**). Interestingly, a trend of reduction of contralateral foot thickness and ankle joint diameter was also observed when comparing the *Vamp3* ^Δmyel^ mice to controls (**Figures 3C, D**).

### Myeloid *Vamp3* deletion reduced pro-inflammatory factors in mice following CFA administration

**Figure 4:**
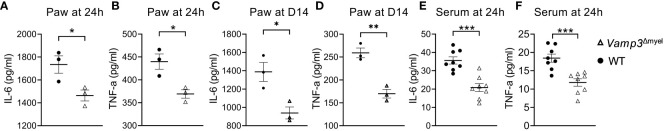
Effect of myeloid *Vamp3* deletion on CFA-induced cytokine production. **(A-D)** Graphs show the amount of IL-6 **(A, C)** and TNF-α **(B, D)** in the CFA injected paws at day 1 **(A, B)** and day 14 **(C, D)** post CFA administration. **(E, F)** Graphs show the amount of IL-6 **(E)** and TNF-α **(F)** in the serum of WT and *Vamp3* ^Δmyel^ mice at 24 hours post CFA injection. Data are presented as mean ± SEM, n≥3 as indicated in the graph. *p<0.05, **p < 0.01, ***p < 0.001; unpaired two-tailed Student’s t-test.

To investigate the underlying mechanism of the reduced inflammation and pain in myeloid Vamp3 deficiency mice, we compared the TNF-α and IL-6 levels in the injected paw and serum of control mice to *Vamp3* ^Δmyel^ mice. IL-6 and TNF-α expression in the CFA injected paws were significantly reduced in *Vamp3* ^Δmyel^ mice vs controls at day 1 (**Figures 4A, B**) and day 14 (**Figures 4C, D**) post CFA injection. Consistently, serum levels of IL-6 and TNF-α at day 1 were also significantly lower in *Vamp3* ^Δmyel^ mice vs controls (**Figures 4E, F**). At day 14, the average serum levels of TNF-α in WT mice and *Vamp3* ^Δmyel^ mice were reduced to 8.1 ± 0.6 pg/ml and 4.9 ± 0.4 pg/ml, respectively (n=8, p<0.001), whereas IL-6 levels were reduced to 12.2 ± 0.6 pg/ml and 8.6 ± 0.5 pg/ml (n=8, p<0.001), respectively. Our results demonstrated that Vamp3 deficiency in myeloid cells decreased TNF-α and IL-6 production in CFA-injected mice.

**Figure 5:**
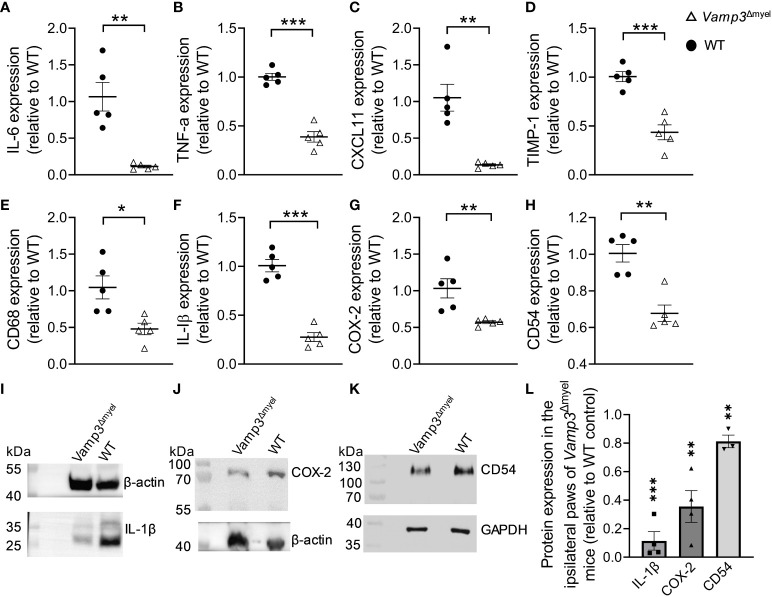
Myeloid *Vamp3* deletion reduced inflammation and pain-related gene expression. **(A-H)** In comparison to WT mice, RT-qPCR showed the downregulation of *IL-6* **(A)**, *TNF-α* **(B)**, *CXCL11* **(C)**, *TIMP-1* **(D)**, *CD68* **(E)**, *IL-1β* **(F)**, *COX-2* **(G)**, and *CD54* **(H)** gene transcription levels in the ipsilateral plantar skins of *Vamp3^Δmyel^* mice at day 14 after CFA injection. **(I-K)** CFA-injected paws of WT and *Vamp3* ^Δmyel^ mice were dissected and homogenized in RIPA lysis and extraction buffer. The extracts were subjected to SDS-PAGE and Western Blotting. IL-1β **(I)**, COX-2 **(J)**, and CD54 **(K)** proteins were detected using their specific antibodies. As indicated, GAPDH or β-actin was probed as an internal loading control. **(L)** The ratios calculated for IL-1β, COX-2, and CD54 and the requisite internal standard (i.e. β-actin or GAPDH) for the CFA-injected paws of *Vamp3* ^Δmyel^ mice were expressed relative to those for WT controls. Data are presented as mean ± SEM, n≥3 as indicated in the graph. *p<0.05, **p < 0.01, ***p < 0.001; unpaired two-tailed Student’s t-test, compared to the WT controls.

We examined gene expression in the paws of control and *Vamp3* ^Δmyel^ mice injected with CFA at day 14, a time point at which CFA had already induced a chronic inflammation phase. RT-qPCR analysis revealed a remarkable downregulation in the transcription levels of inflammation- and pain-associated genes, including *IL-6*, *TNF-α*, *CXCL11*, *tissue inhibitor of metalloproteinases-1* (*TIMP-1*), *CD68, IL-1β, cyclooxygenase-2* (*COX-2*), and *CD54* (**Figures 5A–H**) in the paws of *Vamp3* ^Δmyel^ mice injected with CFA, in comparison to the control group. Western blotting confirmed that the protein expression levels of IL-1β, COX-2, and CD54 in the CFA-injected paws of *Vamp3* ^Δmyel^ mice were significantly reduced when compared to the control mice (**Figures 5I–L**). These findings shed light on the potential role of Vamp3 in regulating the transcription of these crucial genes involved in inflammation and pain processes.

### Vamp3 deficiency in myeloid cells reduced CFA-induced macrophage infiltration and histopathological alterations

**Figure 6:**
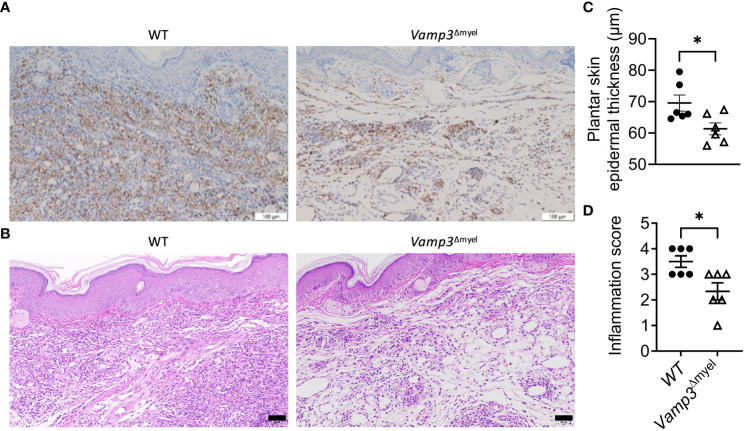
Myeloid Vamp3 deletion reduced macrophage infiltration and inflammation progression in CFA-induced mice. **(A)**, Representative images of immunohistochemical staining of the ipsilateral paw skins of WT and *Vamp3* ^Δmyel^ mice with an anti-CD68 antibody at day 14 following CFA injection. Scale bars, 100 µm. **(B)**, Representative images of the ipsilateral paw skin tissues of WT and *Vamp3* ^Δmyel^ mice stained with HE at day 14 after CFA treatment. Scale bars, 50 µm. **(C)**, Analysis of epidermal thickness of the ipsilateral plantar skins of WT and *Vamp3* ^Δmyel^ mice by H&E staining at day 14 after CFA injection. Epidermal thickness was measured in 5 randomly selected fields of view per mouse. **(D)**, Scores of inflammation in the ipsilateral hind paw tissues by H&E staining. Data in **(C, D)** are presented as mean ± SEM, n=6. *p < 0.05; unpaired two-tailed Student’s t-test.

Next, we investigated whether Vamp3 deficiency in myeloid cells has any effect on macrophage accumulation at the inflamed sites. Immunohistochemical staining demonstrated that myeloid *Vamp3* deletion apparently decreased the accumulation of CD68^+^ cells (mainly macrophages) in the CFA-injected paw skins at day 14 (**Figure 6A**). Histopathological examination using H&E staining showed the paws injected with CFA in control mice exhibited significantly more severe inflammation than those from *Vamp3* ^Δmyel^ mice, characterized by extensive infiltration of inflammatory cells (**Figure 6B**). Furthermore, the epidermal thickness of CFA-injected paw skins of *Vamp3* ^Δmyel^ mice was significantly reduced compared with those from WT mice (**Figure 6C**). Semiquantitative analysis of dermal inflammation indicated that the absence of Vamp3 in myeloid cells significantly reduced CFA-induced inflammation (**Figure 6D**).

Our collective data suggest that myeloid Vamp3 deletion can effectively suppress CFA-induced inflammation and pain by mechanistically reducing macrophage infiltration and inflammatory factors.

## Discussion

Macrophages play a crucial role in initiating the innate immune response, acting as a bridge between the innate and adaptive immune systems. However, under chronic inflammation, they can have detrimental effects, leading to the formation of lesions. Previous research has shown that reducing the expression of Vamp3, a v-SNARE (vesicle-associated SNARE protein) essential for vesicle fusion with the cell membrane, leads to a decrease in TNF-α and IL-6 secretion from cultured macrophages (18). However, the exact role of Vamp3 in chronic inflammation remains uncertain. The major aim of the present study was to investigate the specific contribution of myeloid cell Vamp3 in CFA-induced inflammation and pain, with the aim of identifying a new therapeutic target.

In our current study, we observed that the absence of Vamp3 in cultured BMDMs and PMs led to a decrease in the release of TNF-α and IL-6. This highlights that while other members of the Vamp family, including Vamp7 [which has also been observed in macrophages, as noted by (31)], are expressed, they are unable to fully compensate for the role of Vamp3 in mediating the transport and fusion of cytokine-containing vesicles. Consequently, the absence of Vamp3 in myeloid cells resulted in a significant reduction of CFA-induced paw edema and ankle joint swelling when compared to the WT mice. Deletion of myeloid *Vamp3* led to lower protein expression of TNF-α and IL-6 in the injected paws and plasma levels on day 1 and day 14. IL-1β protein expression was also reduced in the injected paws of the *Vamp3* ^Δmyel^ mice. The reduction in epidermal hyperplasia observed in the *Vamp3* ^Δmyel^ mice, when compared to the control mice in the CFA modeling, could align with the proposed involvement of IL-1β-IL-1R signaling in promoting inflammatory responses, especially in the context of epidermal hyperplasia. This correlation mirrors findings in psoriasis, a condition where IL-1β is known to play a pivotal role in initiating inflammation (32). CD54 plays a crucial role in mediating the recruitment of leukocytes from circulation to inflamed sites in response to inflammatory stimulation. Herein, we observed a notable reduction in the transcription and protein levels of CD54 in *Vamp3* ^Δmyel^ mice. A recent study by Omura et al. demonstrated an escalation in M1 macrophage density after intraplantar injection of CFA, while M2 density remained unchanged at the site of inflammation (23). Our immunohistochemical staining experiments provided clear evidence that the knockout of Vamp3 in myeloid cells resulted in a substantial reduction in local macrophage accumulation in the plantar skin following CFA injection. This finding is further supported by the observed downregulation of *CD68* gene transcription in the CFA-injected paw of *Vamp3* ^Δmyel^ mice, emphasizing the crucial role of Vamp3-regulated cytokines in mediating macrophage infiltration. In contrast, the accumulation of MCP8^+^ cells (mainly in basophils and mast cells) and CD11b^+^ cells (mainly in eosinophils and neutrophils) in the CFA-injected paws were not significantly altered in the *Vamp3* ^Δmyel^ mice (data not shown). The level of CXCL11 was found to be elevated in the plasma of early RA patients. Treatment with methotrexate in combination with anti-TNF (certolizumab-pegol) has been shown to reduce plasma CXCL11 levels (33). In line with these observations, our study revealed a reduction in *CXCL11* gene transcription in the CFA-injected paw tissues of the *Vamp3* ^Δmyel^ mice. This decrease in CXCL11 expression may have implications for the migration of Th1 cells into the inflamed synovial tissues. Additionally, we found a reduction in *TIMP-1* gene transcription in the *Vamp3* ^Δmyel^ mice. TIMP-1, traditionally recognized for its inhibitory effects on metalloproteinases, has recently emerged as a potent cytokine involved in immune cell signaling. Clinical evidence suggests a proinflammatory role of TIMP-1 beyond its metalloproteinase inhibitory function (34). Notably, inflammation-induced expression of TIMP-1 has been observed in subsets of macrophages (35). Interestingly, we also found a substantial decrease in TIMP-1 release from cultured BMDMs upon KO of Vamp3 (data not shown). The impact of TIMP-1 on immune cells under inflammatory conditions warrants further investigation. Our studies demonstrate that the injection of CFA also led to the development of inflammatory edema in the contralateral paw on day 10 post-injection. This corroborates earlier studies that have shown that CFA triggers a biphasic inflammatory response: an initial acute phase marked by localized inflammation in the injected paw on the same day as the injection, followed by a chronic inflammatory phase characterized by inflammatory edema in the contralateral paw, persisting approximately 2 weeks onward (23, 36). The observed reduction in swelling in the contralateral paw may be attributed to a decrease in the release of pro-inflammatory cytokines, particularly IL-6. Taken together, these findings suggest that the diminished inflammation observed in the *Vamp3* ^Δmyel^ mice following CFA treatment can be attributed to a decreased recruitment of macrophages, as well as the suppression of inflammatory cytokine production and secretion.

**Figure 7:**
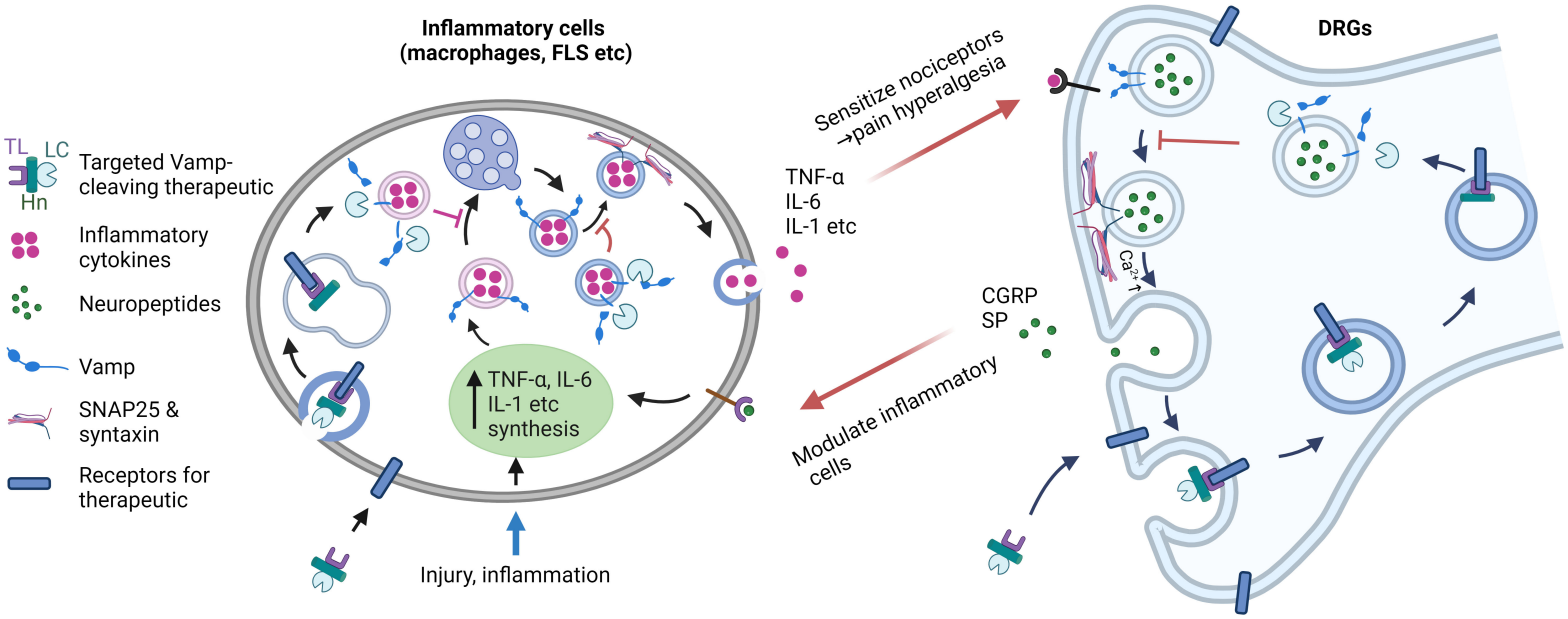
Targeting Vamps expressed in inflammatory cells and sensory neurons offers a strategic approach for effectively suppressing inflammation and pain. During injury and inflammation, proinflammatory cytokines (such as TNF-α, IL-6, etc.) are synthesized and undergo intracellular trafficking and secretion, a process facilitated by Vamp3. The released inflammatory mediators then activate receptors on sensory nerves, leading to the local release of pain peptides (such as substance P and CGRP) mediated by Vamp1. Additionally, pain-related receptors and ion channels are upregulated on the neuronal surface. In chronic diseases, this cascade likely plays a role in the development of hyperalgesia and allodynia. By utilizing protein engineering of Vamp-cleaving botulinum neurotoxin and replacing its native binding domain with a ligand specific for inflammatory cells and sensory neurons, selective inactivation of Vamps in these cells can be achieved. This targeted approach effectively inhibits cytokine and neuropeptide release and normalizes the expression of pain-related receptors and channels, ultimately reducing the enhanced communication between sensory neurons and inflammatory cells. Harnessing such therapeutic properties of novel therapeutics holds significant potential for treating chronic inflammation and associated pain. TL, targeting ligand; LC, light chain Vamp-cleaving protease; Hn, translocation domain of botulinum neurotoxin. Image was created with BioRender.com.

We compared the thermal and mechanical nociceptive thresholds in WT and *Vamp3* ^Δmyel^ mice following CFA injection to assess pain sensitivity over time. Our findings revealed that the *Vamp3* ^Δmyel^ mice exhibited decreased sensitivity to heat stimuli and, to a lesser extent, mechanical stimuli. Furthermore, we observed reduced transcription and protein expression levels of the *COX-2* gene in the CFA-injected paws of the *Vamp3* ^Δmyel^ mice compared to the control mice. In RA, COX-2 is known to be upregulated, promoting inflammation, pain, and joint damage. COX-2 is expressed by inflammatory cells (such as macrophages) and can be induced by TNF- α. TNF-α is implicated in the pathogenies of inflammatory and neuropathic pain, causing long-term mechanical allodynia in both naïve and nerve injury models (37). Our previous study demonstrated that TNF-α enhances the trafficking of transient receptor potential (TRP) V1 (TRPV1) channel to the surface of sensory neurons, a process regulated by SNARE-mediated fusion of large dense-core vesicle fusion with plasmalemma (38). While TRPV1 in peripheral nerve endings is crucial for thermal hyperalgesia, its contribution to CFA-induced mechanical hyperalgesia is relatively minor (39). This may account for the modest effect observed in the *Vamp3* ^Δmyel^ mice in mitigating CFA-induced mechanical hyperalgesia. Nevertheless, the reduced production of these inflammatory cytokines via myeloid *Vamp3* deletion likely contributes to the observed alleviation of CFA-induced inflammatory pain. This, in turn, contributes to the partial improvement in spontaneous mouse locomotor activity, especially within the first 2 weeks after CFA injection. Collectively, our findings underscore the significant role of myeloid Vamp3 in CFA-induced inflammation and pain (**Figure 7**).

It is noted that the reduction of inflammation and relief of pain by myeloid Vamp3 deletion were not complete in this study, implying that myeloid Vamp3 is not the sole contributor. The expression of Vamp3 in other immune cells and/or inflammatory cells may also play a role. Our recent research has indicated that Vamp3, in conjunction with its SNARE partners (SNAP-29 and syntaxin 4), is involved in releasing various inflammatory mediators, including TNF-α, IL-1β, IL-6, IL-17A, EMMPRIN, GM-CSF, and MMP9, from human keratinocytes (40). Th17 cells have also been implicated in RA. IL-6 is an important cytokine in driving the Th17 subset. Additionally, dendritic cells also require Vamp3 to regulate cytokine secretion (41). In our previous study, we also observed that Vamp3 contributes to the release of IL-6 and TNF-α from human synovial cells (22). Therefore, Vamp3 expressed in immune cells (e.g., macrophages, etc) and non-immune cells, such as fibroblast-like synoviocytes associated with inflammatory disease, has become an apparent target. Local inhibition of Vamp-regulated secretory pathways to block the release of multiple critical cytokines, thus halting inflammation, may offer significant advantages over therapies that target individual post-released cytokines or their receptors.

It is worth noting that Vamp1, 2, and 3 isoforms are susceptible to long-acting botulinum neurotoxin (BoNT) serotypes, including B, D, F, G, and X, with type X additionally capable of cleaving Vamp4 and Vamp5 (42). BoNTs are the most powerful inhibitor of neurotransmitter exocytosis and are widely used to treat hyperexcitability disorders in cholinergically innervated muscles or glands. BoNTs can also reduce joint pain severity and improve function (43). While pro-inflammatory cytokine-releasing cells lack the necessary receptors required for BoNTs to enter and act, the modular structure of these neurotoxins presents the possibility of expanding their therapeutic applications through protein engineering, particularly in the inhibition of cytokines and pain transmitters. BoNTs are ~150 kDa proteins composed of a ~50 kDa SNARE-cleaving protease light chain (LC) and a ~100 kDa heavy chain (HC) linked together via a non-covalent disulfide bond. The C-terminal half of the HC (Hc) binds to gangliosides and synaptic vesicle protein 2 (BoNT/A,/D,/E and/F) or synaptotagmin I/II (BoNT/B and/G), determining their neurotropism. The N-terminal half of HC (Hn) forms a channel that allows the protease to cross the endosomal membrane into the cytosol, where the LC truncates the SNAREs necessary for vesicle fusion and the release of neurotransmitter content. The Vamp-cleaving BoNT-protease is the most appropriate constituent of a potential therapeutic for treating RA due to Vamp3 and Vamp1 being essential for exocytosing cytokines and pain-peptides (such as CGRP and substance P etc), respectively, from the abovementioned proinflammatory cells and sensory neurons (44, 45). To achieve targeted delivery of Vamp cleaving protease to block cytokine and neuropeptide release, protein engineering technology can be employed to replace the neuronal acceptor binding domain of BoNT with ligands that bind selectively and specifically to receptors expressed on inflammatory cells and sensory (but not motor) neurons (**Figure 7**). In this regard, prior studies have confirmed that IL-1 receptors are expressed on macrophages, fibroblast-like synoviocytes and pain-related sensory neurons (15, 20, 38, 46–49). Ligands for IL-1 receptors, such as IL-1 agonist (IL-1β) and IL-1 receptor antagonist (IL-1RA), can be selected as a targeting ligand for the delivery of Vamp cleaving protease.

In conclusion, our data in this study demonstrated for the first time that myeloid cell Vamp3 plays a crucial role in CFA-induced inflammation and pain hypersensitivity in mice. Targeted delivery of long-acting Vamp-cleaving BoNT protease into inflammatory cells and sensory neurons could revolutionize the treatment of chronic inflammation conditions by not only attenuating the secretion of pro-inflammatory cytokines but also pain-peptides. Most importantly, the additional latter activity incorporated would result in a reduction in inflammation by blocking neuronal communication with inflammatory cells (see above), thereby blocking the inflammation feed-forward loop (**Figure 7**). Thus, our study opens up a new avenue for the development of novel locally applied, long-acting therapeutics with anti-inflammation and anti-nociceptive potential.

## Data availability statement

The original contributions presented in the study are included in the article/**Supplementary Material**. Further inquiries can be directed to the corresponding authors.

## Ethics statement

The animal study was approved by the Animal Ethics Committee of Henan University, China. The study was conducted in accordance with the local legislation and institutional requirements.

## Author contributions

XD, LL, XY, QF, and RW conducted experiments. WZ, WC, and YL analyzed data. XD, JM, and JW designed research studies, analyzed data, and wrote the manuscript. All authors contributed to the article and approved the submitted version.
