# Supplementary material for: Myeloid Vamp3 deletion attenuates CFA-induced inflammation and pain in mice via ameliorating macrophage infiltration and inflammatory cytokine production
Source: Front Immunol. 2023 Oct 27;14:1239592. doi: 10.3389/fimmu.2023.1239592 (PMC10641732; doi:10.3389/fimmu.2023.1239592)
Supplement: Supplementary file 1 [file DataSheet_1.pdf]

Supplementary Table 1. Nucleotide sequences of the primers used for the RT-qPCR

Gene ID (Accession No.)	Primer sequence (5' to 3')
Mouse IL-6 (NM_031168)	Forward: TACCACTTCACAAGTCGGAGGC
	Reverse: CTGCAAGTGCATCATCGTTGTTC
Mouse TNF-α (NM_013693)	Forward: GTTCTATGGCCCAGACCCTCAC
	Reverse: GGCACCACTAGTTGGTTGTCTTTG
Mouse IL-1β (NM_008361)	Forward: TCCAGGATGAGGACATGAGCAC
	Reverse: GAACGTCACACACCAGCAGGTTA
Mouse CXCL11 (NM_019494)	Forward: CCGAGTAACGGCTGCGACAAAG
	Reverse: CCTGCATTATGAGGCGAGCTTG
Mouse COX-2 (NM_011198)	Forward: GCGACATACTCAAGCAGGAGCA
	Reverse: AGTGGTAACCGCTCAGGTGTTG
Mouse CD68 (NM_009853)	Forward: GGCGGTGGAATACAATGTGTCC
	Reverse: AGCAGGTCAAGGTGAACAGCTG
Mouse CD54 (NM_010493)	Forward: AAACCAGACCCTGGAACTGCAC
	Reverse: GCCTGGCATTTCAGAGTCTGCT
Mouse TIMP-1 (NM_011593)	Forward: TCTTGGTTCCCTGGCGTACTCT
	Reverse: GTGAGTGTCACTCTCCAGTTTGC
Mouse GAPDH (NM_008084)	Forward: CATCACTGCCACCCAGAAGACTG
	Reverse: ATGCCAGTGAGCTTCCCGTTCAG

Primers used in genotyping

Primer Name	Primer sequence (5' to 3')
*Vamp*3-flox-Forward	TCTCCTGTGCAGTTTTAGTCTGTG
*Vamp*3-flox-Reverse	GGTGGACACCATTACCATCTTCAT
LyzM-Cre-Forward	CCCAGAAATGCCAGATTACG
LyzM-Cre-Reverse	CTTGGGCTGCCAGAATTTCTC

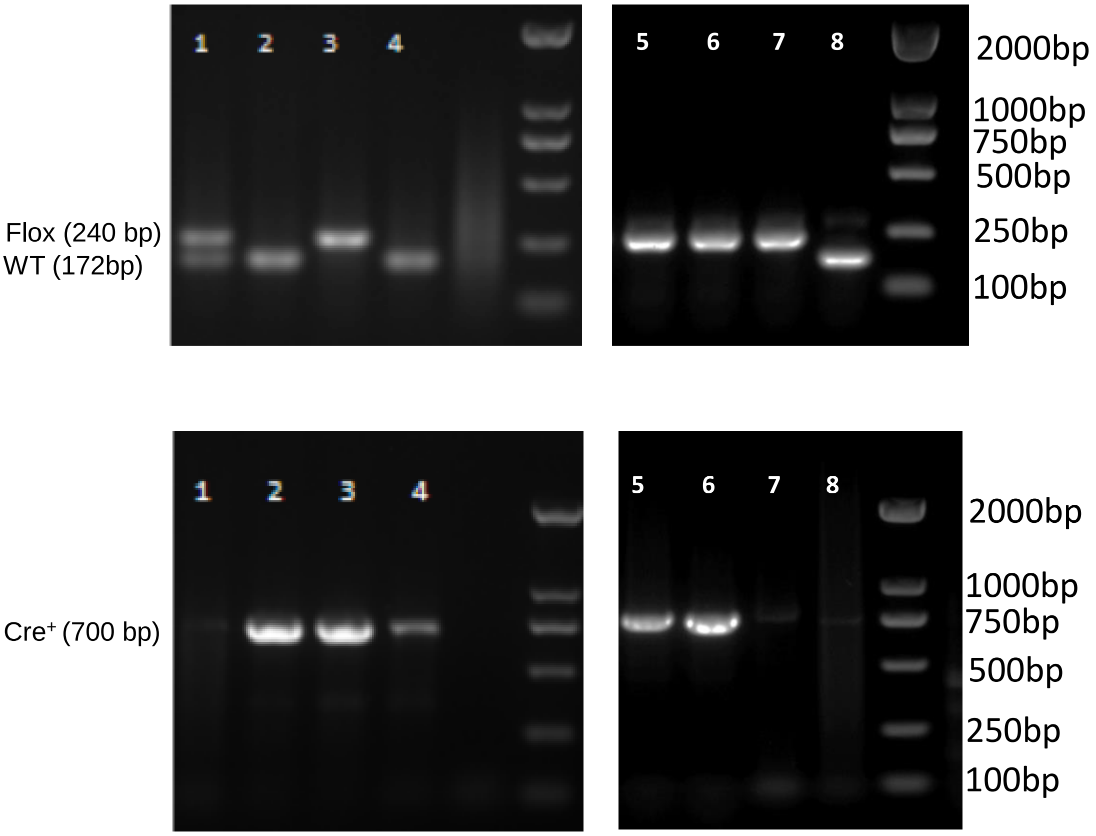

Supplementary Figure 1: Genotyping of *Vamp3*$^{\Delta myel}$ and *LysM-Cre* mice. In the upper panel, the primers used for genotyping are listed. In the bottom panel, a typical PCR was performed to screen mouse genotype. Mouse 1: *Vamp3*$^{flox+/-}$-*Cre*$^{-}$; 2,4: *Vamp3*$^{flox-/-}$-*Cre*$^{+}$; 3, 5, 6: *Vamp3*$^{flox+/+}$-*Cre*$^{+}$; 7: *Vamp3*$^{flox+/+}$-*Cre*$^{-;}$ 8: WT

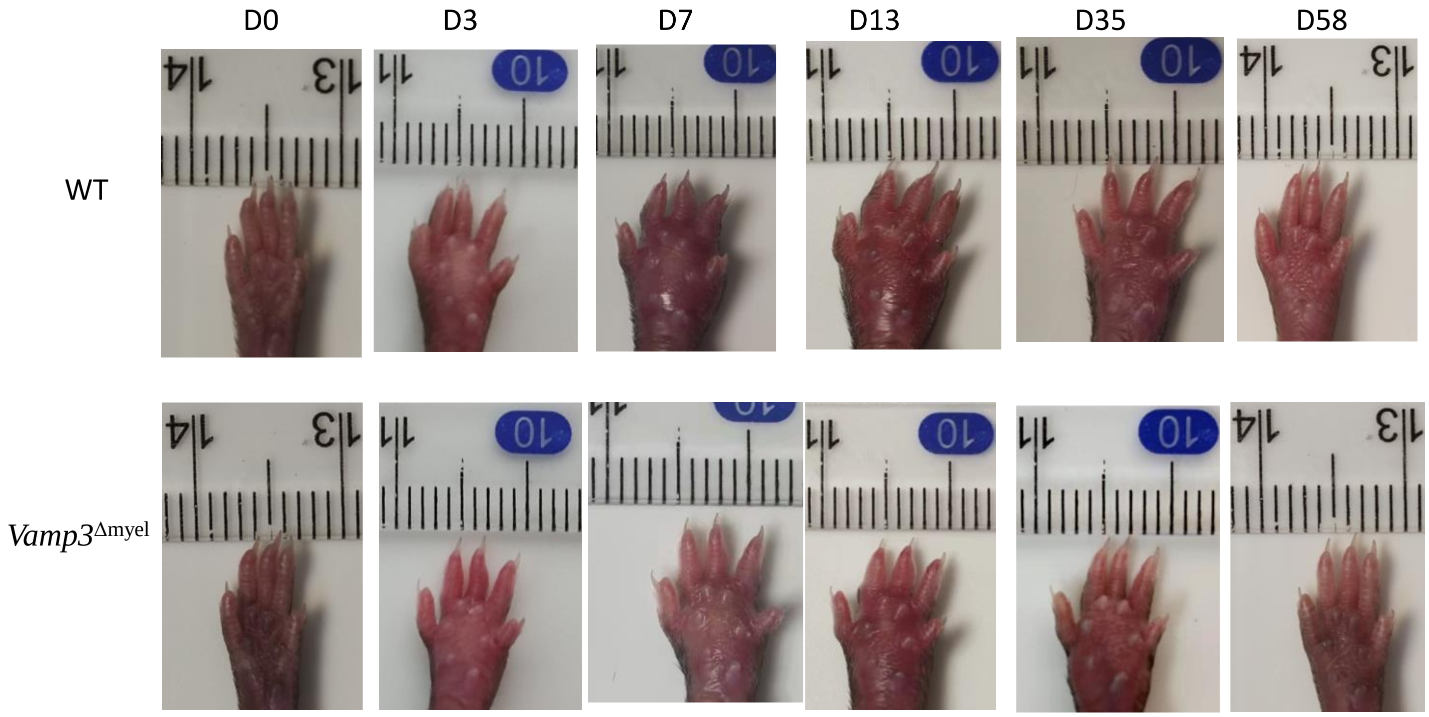

Supplementary Figure 2: The representative photographs show the plantar surface of the ipsilateral hind paw of one *Vamp3*$^{\Delta myel}$ mouse and one WT mouse over time after CFA injection.
